# Translational values of tissue‐resident memory T cells in chronic inflammation and cancer

**DOI:** 10.1002/ctm2.70516

**Published:** 2025-11-25

**Authors:** Wanxin Duan, Xiangdong Wang

**Affiliations:** ^1^ Department of Pulmonary and Critical Care Medicine Zhongshan Hospital Fudan University Shanghai Medical College Shanghai Institute of Clinical Bioinformatics Fudan University Center of Clinical Bioinformatics Shanghai China

1

T cells are central orchestrators of adaptive immunity and play important and complex roles in chronic inflammation, despite that their roles remain even paradoxical. The dysregulations of T cells occur in chronic diseases, such as inflammation and cancer, from being protectors to potent drivers of tissue pathology.^1^, ^2^, ^3^ Of those, the pro‐inflammatory tissue‐resident memory (TRM) T cells accumulate within the tissue, perpetuating a cycle of inflammation. Subsets of TRM T cells, including those producing the highly inflammatory cytokine interleulin‐17 (IL‐17), are directly implicated in tissue damage, to form the ectopic lymphoid tissues, remodel the microenvironment, and amplify the local response in inflammation and cancer.^4^, ^5^ Reformed lymphoid alter local gradients of inflammatory mediators to trap and retain more lymphocytes and exacerbate the microenvironmental bioecology. The pre‐activated TRM‐like T cells harboured in lungs of smokers as the pre‐existing state of a tissue can create an immune pressure that reprograms subsequent tumour evolution and response to therapy and profoundly influences disease progression.^6^

The deep understanding of TRM T‐cell phenomes and bio‐behaviours provides new insights for the identification of diagnostic biomarkers and therapeutic targets. The TRM T cells as a special type of memory T cells are categorised on basis of the locations (e.g., gut‐TRM, lung‐TRM, brain‐TRM), cell surface antigens (e.g., CD8⁺TRM, CD4⁺TRM), or cell identity gene markers measured by single‐cell RNA sequencing (scRNA‐seq).^7^, ^8^, ^9^ One of biological characteristics is their long‐term residence in specific tissue to take an immediate action in the initiation of immune responses to invaded pathogens and reduction infectious spreads, faster than circulating memory T cells. Of those, CD8⁺TRM T cells are the majority responsible for antiviral and anti‐tumour immunity and can directly terminate infected cells and pathogen replication by releasing inflammatory mediators and enzymes. CD4⁺TRM can support other immune cells (like B cells for antibody production, macrophages for activation), and regulate local immune responses to infectious and autoimmune diseases by enhancing the synergistic effects of the immune networks. In addition, TRM T cells play critical roles in the tissue repair by controlling microenvironmental contents of inflammatory mediators and recognising abnormal cells like infected cells or cancer cells to reduce the risk of tissue damage and maintain microenvironmental immune bioecology. The molecular processes of reservable immune memory in TRM T cells can provide a number of alternatives for vaccination and immunotherapy.

Recent redefinition of redefining T‐cell behaviour in inflamed or tumour microenvironment are largely driven by high‐resolution techniques such as scRNA‐seq, spatial transcriptomics and multi‐omics integration. Novel T‐cell subsets/states are re‐uncovered using scRNA‐seq, different from the descriptions using bulk RNA analyses.

The formation of TRM T cells in inflamed and injured tissues is regulated by multiple factors. The functionally distinct TRM subsets follow divergent developmental paths, for example, IFN‐γ‐producing TRM1 cells depend on a T‐bet‐Hobit axis, whereas IL‐17‐producing TRM17 cells are programmed independently by the transcription factor c‐Maf.^10^ This highlights a remarkable degree of tissue‐specific specialisation within TRM lineages. Additionally, external factors such as chemical sensing and metabolic cues significantly influence TRM cell behaviour. The transcription factor C/EBPβ as a sensor for certain chemicals can directly promote T‐cell‐driven intestinal inflammation,^11^ while the distinct population of Granzyme K‐expressing CD8+ T cells can be a key driver for the recurrence of chronic rhinosinusitis.^12^ Critically, cellular metabolism has emerged as a central regulatory hub. The enzyme ATP citrate lyase is indispensable for T‐cell‐driven colitis by changing the production of glycolytic ATP and the biosynthesis of phospholipids and phosphatidylcholine.^13^ TRM T‐cell survival depends on exogenous lipid uptake and the metabolic byproduct lactate can actively reprogram T cells in inflamed tissues. By producing acetyl‐CoA, ACLY provides the essential substrate for histone acetylation at pro‐inflammatory gene loci, thereby epigenetically promoting the expression of cytokines such as IFN‐γ and IL‐17A.

The molecular phenomes and regulations of TRM T cells are also highlighted in clinical and translational discovery and medicine. Using scRNA‐seq, lung tissue CD8+naïve and memory T cells were found to participate in the differentiation of CD8+T cells to exhausted and/or cytotoxic cells and positively regulate cell death and cytokine production in patients with multiple primary lung cancer.^14^ Stem cell‐like memory T cells, a population of long‐lived memory T cells with the capacity for self‐renewal and differentiation, reduced in newly diagnosed multiple myeloma.^15^ The CD8+TRM T cells with high expression of inhibitory molecules were noticed in bone marrow of patients with amyloid light chain at diagnosis and quickly activated with downregulation of suppressive molecules and upregulation of IFNG expression after a combination of daratumumab with cyclophosphamide, bortezomib and dexamethasone. These cells were rapidly activated, showing reduced expression of suppressive markers and increased IFNG transcription.^16^ In the lung, TRM T cells have also been shown to interact with stromal.^16^ Lung TRM T cells were also found to closely communicate with the interstitial cells like telocytes, to maintain the activation of tissue repair.^17^ It indicates that molecular bio‐behaviours o TRM T cells can be a source of diagnostic biomarker discovery, and also of therapeutic target identification. However, the clinical application of TRM T‐cell‐based diagnostics and therapies requires further refinement of their molecular signatures. The accuracy and specificity of TRM T‐cell identity gene mark panels for their subsets and functional states should be furthermore defined and standardised on basis of tissue types and diseases, to meet the requests for clinical application and improve the outcomes of patients.^18^

Look forward, the biological function and protective effects of TRM T cells highly dependent upon the tissue specificity and heterogeneity of TRM T‐cell origins and differentiation trajectories. The spatiotemporally localisations of intra‐ and extra‐cellular signaling can orient the TRM T cells to differentiated or progenitor‐like, regulated by distinct ligand‒receptor activities, cytokine gradients and specialised cellular contacts through the multiple signal pathways such as TGFβ or CXCL9 and CXCL10.^19^ Furthermore, the real live microenvironment of TRM T cells is stereologically spatiotemporal, dynamical and real‐time changeable. The continuous spatial transcriptomes can provide stereological images of TRM T cells for visualisation of multi‐dimensional connections/interactions, while the stereo‐cell sequencing can provide the dynamic and morphological platform to investigate how TRM T cells regulate the formation of inflamed and cancer microenvironment.^20^, ^21^ With the development of multiomics, the artificial intelligent TRM T single cell will be constructed to provides reliable and rapid information for understanding the dynamics of molecular regulations and impacting clinical diagnoses and prediction of the disease at the single‐cell level.^22^ Thus, the deeper mining TRM T‐cell functions, specificities and regulations among their subsets, interacted cells and locations will create more alternatives of clinical therapies.
